# Impact of Zinc and/or Herbal Mixture on Ruminal Fermentation, Microbiota, and Histopathology in Lambs

**DOI:** 10.3389/fvets.2021.630971

**Published:** 2021-01-28

**Authors:** Daniel Petrič, Dominika Mravčáková, Katarína Kucková, Svetlana Kišidayová, Adam Cieslak, Malgorzata Szumacher-Strabel, Haihao Huang, Pawel Kolodziejski, Anna Lukomska, Sylwester Slusarczyk, Klaudia Čobanová, Zora Váradyová

**Affiliations:** ^1^Institute of Animal Physiology, Centre of Biosciences of the Slovak Academy of Sciences, Košice, Slovakia; ^2^Department of Animal Nutrition, Poznan University of Life Sciences, Poznan, Poland; ^3^Department of Animal Physiology, Biochemistry and Biostructure, Poznan University of Life Sciences, Poznan, Poland; ^4^Department of Preclinical Sciences and Infectious Diseases, Poznan University of Life Sciences, Poznan, Poland; ^5^Department of Pharmaceutical Biology and Botany, Wroclaw Medical University, Wroclaw, Poland

**Keywords:** bacteria, ciliated protozoa, hematological profiles, histology, phytochemicals, sheep

## Abstract

We investigated the effect of diets containing organic zinc and a mixture of medicinal herbs on ruminal microbial fermentation and histopathology in lambs. Twenty-eight lambs were divided into four groups: unsupplemented animals (Control), animals supplemented with organic zinc (Zn, 70 mg Zn/kg diet), animals supplemented with a mixture of dry medicinal herbs (Herbs, 100 g dry matter (DM)/d) and animals supplemented with both zinc and herbs (Zn+Herbs). Each lamb was fed a basal diet composed of meadow hay (700 g DM/d) and barley (300 g DM/d). The herbs *Fumaria officinalis* L. (FO), *Malva sylvestris* L. (MS), *Artemisia absinthium* L. (AA) and *Matricaria chamomilla* L. (MC) were mixed in equal proportions. The lambs were slaughtered after 70 d. The ruminal contents were used to determine the parameters of fermentation *in vitro* and *in vivo* and to quantify the microbes by molecular and microscopic methods. Samples of fresh ruminal tissue were used for histopathological evaluation. Quantitative analyses of the bioactive compounds in FO, MS, AA, and MC identified 3.961, 0.654, 6.482, and 12.084 g/kg DM phenolic acids and 12.211, 6.479, 0.349, and 2.442 g/kg DM flavonoids, respectively. The alkaloid content in FO was 6.015 g/kg DM. The diets affected the levels of total gas, methane and *n-*butyrate *in vitro* (*P* < 0.046, < 0.001, and < 0.001, respectively). Relative quantification by real-time PCR indicated a lower total ruminal bacterial population in the lambs in the Zn and Zn+Herbs groups than the Control group (*P* < 0.05). The relative abundances of *Ruminococcus albus, R. flavefaciens, Streptococcus bovis*, and *Butyrivibrio proteoclasticus* shifted in the Zn group. Morphological observation found a focally mixed infiltration of inflammatory cells in the lamina propria of the rumen in the Zn+Herbs group. The effect of the organic zinc and the herbal mixture on the parameters of ruminal fermentation *in vitro* was not confirmed *in vivo*, perhaps because the ruminal microbiota of the lambs adapted to the zinc-supplemented diets. Long-term supplementation of a diet combining zinc and medicinal herbs, however, may negatively affect the health of the ruminal epithelium of lambs.

## Introduction

The ruminal microbial fermentation of dietary substrates plays a main role in the ability of ruminants to use fibrous dietary substrates but is also associated with emissions of methane and the excessive excretion of nitrogen in manure. Modern animal production systems require the maintenance of optimal animal health and the safe and efficient production of high-quality animal products. Understanding the ruminal microbiome in all aspects of bacterial, archaeal or eukaryotic populations and in all factors for manipulating the microbiome to maximize productivity while decreasing negative environmental impacts is therefore necessary (1, 2).

Our previous results indicated that replacing 10% of meadow hay with different mixtures of dry medicinal plants could influence the patterns of fermentation (3, 4). Recent findings with plant nutraceuticals highlight the dependence of the effect of dry medicinal plants on the variety and synergy of plant polyphenols and the combination of bioactive compounds, which together affect and contribute to a specific pharmacological efficacy (5, 6). Zinc plays catalytic, structural and regulatory roles for enzymes, proteins and transcription factors and is thus a key trace element for improving immunological functions (7, 8). The bioavailability of zinc in the diets of ruminants also depends on the chemical form, content and interaction of zinc with dietary constituents (9, 10). The most recent requirements and recommendations for dietary zinc in ruminants vary between 40 and 130 mg/kg DM of the complete diet (11). The organic forms of trace elements bound by organic ligands should be more resistant to interactions in the ruminant digestive tract and can be more bioavailable than inorganic sources (12). The rumen of ruminants allows the selective uptake of nutrients generated by intraruminal microbial fermentation because the rumen is covered by a stratified epithelium that consists of leaflike papillae, which greatly increase the area and size of the absorptive surface (13). Epithelial surfaces are complex chemical and biological barriers that prevent the invasion of microbes or other potentially harmful pathogens, but they also harbor many beneficial microorganisms (14).

Our recent *in vitro* study reported that a mixture of fumitory, mallow, wormwood and chamomile possessed a strong ruminal antioxidant capacity with the potential for inducing desirable changes in the gastrointestinal ecosystem during ruminant fermentation (15). Ruminal volatile fatty acids (VFAs) arise mostly from the fermentation of dietary carbohydrates and are absorbed through the ruminal epithelium; we therefore hypothesized that this herbal mixture, together with organic zinc, would affect not only ruminal fermentation and the microbial population but also ruminal histopathology. Our knowledge is also based on our previous findings indicating that a combination of zinc and a special medicinal herbal mixture can positively influence the health of lambs infected with gastrointestinal nematodes (16). We investigated the effects of dietary supplements containing organic zinc and a mixture of medicinal herbs (*Fumaria officinalis* L., *Malva sylvestris* L., *Artemisia absinthium* L., and *Matricaria chamomilla* L.) on ruminal fermentation, the microbial population and the histopathology of the lambs.

## Materials and Methods

### Lambs, Diets, and Experimental Design

The experimental design followed the standards of the European Union for the protection of animals under European Community guidelines (EU Directive 2010/63/EU). The Ethical Committee of the Institute of Animal Physiology of the Centre of Biosciences of the Slovak Academy of Sciences approved the experimental protocol (resolution no. Ro-4065/18-221/3). Twenty-eight castrated male Improved Valachian lambs ~5 months old with body weights of 22.6 ± 2.94 kg were housed individually in pens for 30 d for acclimatization to feeding with free access to water. The animals were divided into four groups (*n* = 7) based on their live weights. The experimental treatments were as follows: (a) a basal diet (Control) composed of 700 g DM/d meadow hay and 350 g DM/d ground barley; (b) a basal diet enriched with a zinc chelate of amino acids hydrate (Zn, 70 mg Zn/kg of diet/d); (c) a basal diet enriched with a mixture of herbs (Herbs, 100 g DM/d); and (d) a basal diet enriched with a combination of zinc and the mixture of herbs (Zn+Herbs). Aliquots of zinc Availa-Zn 100 EU (Zinpro Corporation, Eden Prairie, USA) were directly mixed with the ground barley. The mixture of herbs (AGROKARPATY, Plavnica, Slovak Republic) contained 33% each of *F. officinalis* (FO), *M. sylvestris* (MS), and *M. chamomilla* (MC) and 1% *A. absinthium* (AA). All experimental groups received diets for 70 d. The lambs were fed twice daily at the same time each day. Before the feeding on the next day, the feed refused by each lamb was sampled and weighed. All samples of refused feed were composited at the end of the study for each lamb and stored at −20°C for later analysis to evaluate nutrient intake. The lambs were weighed at the beginning of the study and on day 35 (D35) and D70. Samples of blood were collected on D0, D35, and D70 from the jugular vein of each lamb using a 21-gauge needle and syringe and deposited into microtubes containing 1.6 mg/mL EDTA-K3 (Sarstedt AG & Co, Nümbrecht, Germany). Hematological parameters were immediately determined using an Abbott CELL-DYN 3700 automated hematological analyzer (Global Medical Instrumentation, Inc., Ramsey, USA).

All animals were killed according to European Commission rules (Council Regulation 1099/2009) for slaughtering procedures (17) at the end of the experiment on three consecutive days (at the abattoir of the Centre of Biosciences of SAS, Institute of Animal Physiology, Košice, Slovakia, No. SK U 06018). The carcasses were sent to the Department of Pathological Anatomy and Pathological Physiology, University of Veterinary Medicine and Pharmacy in Košice, Slovak Republic. Ruminal contents were collected, and samples of fresh ruminal tissues were fixed in 10% neutral buffered formalin.

### *In vitro* Experiment

The experiment was carried out using the *in vitro* gas production technique (IVGPT) on batch-culture incubations of buffered ruminal fluid (RF) incubated at 39°C for 24 h under anaerobic conditions (18). The ruminal contents were collected separately from each lamb of each treatment immediately after the slaughter in the abattoir, packed in prewarmed flasks and transported to the laboratory as was previously described (3). The ruminal contents were forced through four layers of cheesecloth and pooled in equal volumes based on the dietary treatments of the donor animals. The pooled RF was purged with CO_2_, mixed with McDougall's buffer (19) in a 1:1 ratio and dispensed in volumes of 35 mL into fermentation bottles (120 mL) containing 250 mg (DM basis) of a substrate. Meadow hay (MH) and barley grain (BG) were used as the basic components of the diet (700:300, w/w) with the use of additive zinc (0.025 g/bottle), herbs (0.025 g/bottle) or both, respectively. Herbs, MH and BG were ground using a grinder (Molina, MIPAM, Ceské Budějovice, Czech Republic) and sieved through 0.15–0.40 mm screens. The *in vitro* experiment was arranged in a completely randomized design using the four diets (Control, Zn, Herbs, and Zn+Herbs) in fermentations with the four inocula of ruminal fluids (Control, Zn, Herbs and Zn+Herbs), with three replicates (three incubation bottles) for each diet and inoculum. The experiment was repeated three times within three consecutive days (*n* = 3 × 3).

### Chemical Analysis and Measurements

The dietary substrates were analyzed in triplicate using standard procedures for dry matter (method no. 967.03), nitrogen (method no. 968.06), crude protein (method no. 990.03) and ash (method no. 942.05) (20). The contents of acid-detergent fiber (ADF) and neutral detergent fiber (NDF) were determined (21) using the FiberCap™ 2021/2023 system (FOSS Analytical AB, Höganäs, Sweden). NDF in the forages was assayed without a thermally stable amylase and was expressed inclusive of residual ash. NDF in the concentrate was assayed using a thermally stable amylase and was expressed inclusive of residual ash. ADF was also expressed inclusive of residual ash. The chemical compositions of the dietary substrates are presented in Table 1.

**Table 1:** Chemical compositions (g/kg DM) of the dietary ingredients.

**Item**	**Meadow hay**	**Barley**	**Herbs^**a**^**	**Availa-Zn100 EU^**b**^**	**Barley + Availa-Zn100^**c**^**
Dry matter (DM, g/kg)	894	875	874	976	869
Neutral-detergent fiber	535	277	390	286	516
Acid-detergent fiber	345	108	217	237	138
Nitrogen	23	22	33	34	26
Crude protein	144	137	206	212	163
Ash	77	25	127	361	27

a Dry medicinal herbs (AGROKARPATY, Plavnica, Slovak Republic);

b Availa-Zn 100 EU (Zinpro Corporation, Eden Prairie, USA);

c *Zn diet, a mixture of zinc (Availa-Zn 100 EU) and ground barley*.

The volume of accumulated total gas was determined after 24 h using IVGPT. For the analysis of methane, 1 mL of gas was collected using IVGPT and an air-tight syringe (GASTIGHT Syringes, Hamilton Bonaduz AG, Switzerland) and injected into a gas chromatograph. The VFAs and methane were analyzed on a PerkinElmer Clarus 500 gas chromatograph (Perkin Elmer, Shelton, USA) (22). Methane production *in vivo* was calculated based on the stoichiometric relationships between VFA composition and methane production (23). The pHs of the batch cultures were measured using a pH meter (InoLab pH Level 1, Weilheim, Germany). The concentrations of ammonia-N in the inocula were determined using the phenol-hypochlorite method (24).

### Analysis of Flavonoids and Phenolic Acids

Each of the herbaceous materials (100 mg), *F. officinalis, M. sylvestris, A. absinthium*, and *M. chamomilla*, were ground to a fine powder and extracted three times with 80% methanol (MeOH) at 40°C for 30 min. The extracts were evaporated to dryness, dissolved in 2 mL of Milli-Q water (acidified with 0.2% formic acid) and purified by solid-phase extraction using a 60-mg Oasis HLB 3cc Vac Cartridge (Waters Corp., Milford, USA). The Milli-Q water was prepared by an ultrapure water system (Barnstead International, Dubuque, USA). The cartridges were washed with 0.5% MeOH to remove carbohydrates and then washed with 80% MeOH to elute the phenolics. The phenolic fraction was re-evaporated and dissolved in 1 mL of 80% MeOH (acidified with 0.1% formic acid). The sample was then centrifuged at 23,000 × g for 5 min before spectrometric analysis. All analyses were performed in triplicate for three independent samples and stored at −20°C before analysis.

### Analysis of Alkaloids

Herbal materials from FO were ground to a fine powder, and 100 mg were extracted with 0.5M H_2_SO_4_ in an ultra-bath at 25°C for 20 min; the procedure was then repeated, and the filtrates were combined. The filtrates were adjusted to pH 9-10 using 1M NaOH and separated using CHCl_3._ The lower organic layer was collected, evaporated to dryness under reduced pressure and then dissolved in 80% MeOH for further analysis.

### Ultra-High-Resolution Mass Spectrometry (UHRMS)

The bioactive compounds of each medicinal herb (FO, MS, AA and MC) were identified using UHRMS on a Dionex UltiMate 3000RS system (Thermo Scientific, Darmstadt, Germany) with a charged aerosol detector connected to a Compact high-resolution quadrupole time-of-flight mass spectrometer (Bruker Daltonik GmbH, Bremen, Germany). The metabolome of the mixture of herbs was chromatographically separated on a 2.1 × 100 mm, 2.6 μm, Kinetex C18 column (Phenomenex, Torrance, USA), with mobile phase A consisting of 0.1% (v/v) formic acid (FA) in water and mobile phase B consisting of 0.1% (v/v) FA in acetonitrile. A linear gradient from 7 to 30% of phase B in phase A over 20 min was used to separate the phenolic compounds, with a short 0.3 min calibration segment from 0 to 0.5 min. The flow rate was 0.3 mL/min, and the column was held at 25°C. Spectra were acquired in negative-ion mode over a mass range from m/z 100 to 1,500 with a frequency of 5 Hz. The operating parameters of the ESI ion source were: capillary voltage, 3 kV; dry gas flow, 6 L/min; dry gas temperature, 200 °C; nebulizer pressure, 0.7 bar; collision radio frequency, 700.0 V; transfer time, 100.0 μs and pre-pulse storage, 7.0 μs. Ultrapure nitrogen was used as the drying and nebulizer gas, and argon was used as the collision gas. The collision energy was set automatically from 15 to 75 eV depending on the mass of the fragmented ion. The data were calibrated internally using sodium formate introduced into the ion source at the beginning of each separation via a 20-μL loop. The spectra were processed using Bruker DataAnalysis 4.3 software (Bruker Daltonik GmbH, Bremen, Germany). The amounts of the phenolic acids in the samples were calculated as the chlorogenic acid (CAS 327-97-9, 3-caffeoylquinic acid) equivalent, and hyperoside (CAS 482-36-0, quercetin 3-galactoside) was used for calculating the amounts of the flavonoids identified. Stock solutions of hyperoside and chlorogenic acid were prepared in MeOH at concentrations of 3.1 and 4.1 mg/mL, respectively, and kept frozen until used. Calibration curves for these two compounds were constructed based on seven concentration points (from 500 to 3.9 μg/mL).

The total content of alkaloids was determined as the chelidonine (CAS 476-32-4) equivalent from the calibration curves based on seven concentration points of chelidonine (from 200 to 1.2 μg/mL). The alkaloids were separated using the same HPLC conditions as with the phenolic compounds, with one exception: positive-ion mode was used for the acquired spectra in auto MS/MS. All analyses were performed in triplicate.

### Quantification of Ruminal Microbes

Samples for counting ciliate protozoa were fixed in equal volumes of 8% formaldehyde, and the protozoa were counted and identified microscopically as described by Williams and Coleman (25). DNA for quantifying bacteria was isolated from the ruminal samples using a Mini Bead-Beater (BioSpec, Bartlesville, USA) for cell lysis (26), followed by purification using a QIAamp DNA Stool Mini Kit (Qiagen, Hilden, Germany). DNA concentrations and qualities were measured using a NanoPhotometer® NP80 (Implen GmbH, München, Germany). Eubacteria and archaea were quantified by real-time PCR using the PCR primers (27). The relative abundance of the 16S rRNA gene was expressed as an arbitrary unit (AU) relative to the total abundance of bacterial genes of the Control group.

### Histological Parameters

Samples of fresh ruminal tissues were washed in a phosphate buffer (0.1 M, pH 7.4), put in plastic containers and fixed in a 10% buffered FA solution as pieces of tissue spread on flat polystyrene. The fixed material was processed using a series of reagents and embedded in Paraplast PLUS paraffin blocks (Leica, Buffalo Grove, USA), which were then cut with a rotary microtome into sections 3.5 μm thick. Slides with a paraffin section were automatically stained with hematoxylin and eosin (Varistain Gemini Thermo Scientific, Runcorn, UK). An Axio Lab. 1 microscope (Carl Zeiss, Jena, Germany) equipped with a Zeiss Axiocam ERc5s digital camera was used for histological evaluation. Photographs were analyzed and recorded using ZEN 2.3 (blue edition) software (Carl Zeiss Microscopy GmbH, 2011).

### Data Analysis

The data were statistically analyzed using GraphPad Prism 8.3.0 (538) 2019 (GraphPad Software, Inc., San Diego, USA). Data for the parameters of ruminal fermentation and *in vitro* ciliate populations were analyzed using two-way analyses of variance (ANOVAs). The model included effects for diets, inocula and the diet × inoculum interaction. Statistical analysis of the hematological parameters used an ANOVA as a repeated-measure mixed model that represented the four animal groups and the sampling days. *In vivo* data were evaluated by multiple comparisons of one-way ANOVAs using Dunnett's multiple comparisons test. The total and differential counts of the ruminal ciliates were analyzed using the non-parametric Kruskal-Wallis test. The effects were determined to be significant at *P* < 0.05.

## Results

### Phytochemical Substances in the Medicinal Herbs

The phytochemical substances in FO consisted of 12.211 g/kg DM flavonoids, 3.961 g/kg DM phenolic acids (Table 2) and 6.015 g/kg DM alkaloids (Table 3). The phytochemical substances in MS consisted of 6.479 g/kg DM flavonoids and 0.654 g/kg DM phenolic acids (Table 2). The phytochemical substances in AA consisted of 0.349 g/kg DM flavonoids and 6.482 g/kg DM phenolic acids (Table 2). The phytochemical substances in MC consisted of 2.442 g/kg DM flavonoids and 12.084 g/kg DM phenolic acids (Table 2).

**Table 2:** Concentrations of the main bioactive compounds in medicinal herbs (g/kg DM).

**RT (min)**	**UV (nm)**	***m/z*[M-H]^**−**^**	**MS^**2**^**	**MS^**2**^ fragments**	**Formula**	**Compounds**	**Flavonoids**	**Phenolic acids**
***Fumaria officinalis***								
7.00	250/326	295.046	179.0338		C_13_H_12_O_8_	Caffeoylmalic acid		1.212
7.80	227/315	163.0395	119.0499		C_9_H_8_O_3_	O-Coumaric acid		0.742
9.00	255/352	625.1398	301.0337		C_27_H_30_O_17_	Quercetin-O-Hex-Hex	2.384	
9.40	255/352	595.1287	301.0339		C_26_H_28_O_16_	Quercetin O-Pen-Hex	3.500	
9.90	252/351	609.1472	300.0279	285	C_27_H_30_O_16_	Isoquercitrin O-Dhex		0.934
10.20	255/354	463.0882	301.0337		C_21_H_20_O_12_	Quercetin O-Hex	1.706	
10.90	221/329	593.1520	285.0397		C_27_H_30_O_15_	Kaempferol-3-O-rutinoside	0.464	
11.50	255/365	639.1561	315.0504		C_28_H_32_O_17_	Isorhamnetin-O-Hex-Hex	0.558	
						Total contents:	12.211	3.961
***Malva sylvestris***								
7.00	523	757.1846	347.0761	329,261,509	C_32_H_39_O_21_	Delphinidin 5-glucoside 3-lathyroside	1.644	
7.90	308	163.0381	119.0502		C_9_H_8_O_3_	Coumarinic acid		0.468
10.00		609.1458	301.0330		C_27_H_31_O_16_	Quercetin-3-O-rutinoside	0.395	
10.20	268/343	447.0928	285.0386		C_21_H_20_O_11_	Kaempferol-O-Hex	0.494	
11.40	268/336	431.0978	269.0435		C_21_H_20_O_10_	Apigenin-O-Hex	1.560	
						Total contents:	6.479	0.654
***Artemisia absinthium***								
4.10	215/325	353.0877	191.0567	179,161,135	C_16_H_18_O_9_	Chlorogenic acid		3.416
11.00		515.1193	353.0867	191,179,135	C_25_H_24_O_12_	1.5-Dicaffeoylquinic acid		2.124
11.20		653.1719	345.0595	330,302	C_29_H_34_O_17_	Spinacetin 3-rutinoside	0.241	
11.70		515.1192	353.0869	173,179,191,155	C_25_H_24_O_12_	4.5-Dicaffeoylquinic acid		0.610
						Total contents:	0.349	6.482
***Matricaria chamomilla***								
4.30	215/300	353.0877	191.0567		C_16_H_18_O_9_	3-O-Caffeoylquinic acid		1.777
9.00	235/290/319	355.1029	193.049	149	C_16_H_20_O_9_	Methyl 4-O-beta-d-glucopyranosyl caffeate		3.202
9.70	255/354	463.0879	301.0337	151	C_21_H_20_O_12_	Quercetin O-Hex	0.199	
10.30	257	447.0920	285.0386		C_21_H_20_O_11_	Kaempferol O-Hex	1.363	
10.70	217/291/325	515.1189	353.0877	179,191	C_25_H_24_O_12_	3.5-Dicaffeoylquinic acid		0.824
11.00	217/291/325	515.1197	353.0869	191,179	C_25_H_24_O_12_	1.5-Dicaffeoylquinic acid		3.016
11.40	266/300	431.0976	269.0434		C_21_H_20_O_10_	Apigenin O-Hex	0.150	
11.70	215/290/325	515.119	353.0868	173,179,191	C_25_H_24_O_12_	4.5-Dicaffeoylquinic acid		0.851
14.40	218/268/339	473.1085	269.0427	406	C_23_H_22_O_11_	Apigenin -O-(Hex-Ac)	0.210	
						Total contents:	2.442	12.084

**Table 3:** Concentrations of the main alkaloids in *Fumaria officinalis* (g/kg DM).

**RT (min)**	**UV (nm)**	***m/z*[M-H]^**−**^**	**MS^**2**^**	**MS^**2**^ fragments**	**Formula**	**Compounds**	**Alkaloids**
7.70	272	354.1366	305.0811	279,233,323,336	C_20_H_19_NO_5_	Parfumine	0.884
8.40	280	328.1572	265.0865	237,297,313,178	C_19_H_21_NO_4_	Cularimine	0.102
8.50	288	370.1678	291.1029	263,352,337	C_21_H_23_NO_5_	Fumaricine	0.102
8.90	285	326.1410	311.1172	277,294,251,178	C_19_H_19_NO_4_	Cheilanthifoline	0.231
9.20	286	354.1360	275.0713	336,247	C_20_H_19_NO_5_	Chelidonine	0.154
9.40	289	398.1621	338.1397	277,323,249	C_22_H_23_NO_6_	Not determined	0.530
10.20	289	354.1362	275.0692	247,293,206	C_20_H_19_NO_5_	Protopine	0.873
10.60	288	354.1727	206.1139	275,311,338,292	C_21_H_23_NO_4_	Protopine type	0.367
10.80	271	352.1193	279.0647	309,321,263,251	C_20_H_17_NO_5_	Fumariline	1.728
11.20	288	324.1230	249.0764	307,277,219,176	C_19_H_17_NO_4_	Stylopine	0.785
						Total contents:	6.015

### Effect of Dietary Substrates on Ruminal Fermentation *in vitro*

The effects of the dietary substrates and inocula on the parameters of ruminal fermentation *in vitro* are presented in Table 4. The inocula of the donor animals affected the values of all parameters of fermentation (*P* < 0.001). Diet significantly affected total gas (*P* < 0.046) and methane (*P* < 0.001) production. The amount of *n*-butyrate also varied among the dietary treatments (*P* < 0.001), with *n*-butyrate concentrations higher for the Zn and Zn+Herbs groups than the Control group. The different ruminal inocula also significantly affected the protozoal populations of the lambs *in vitro* (Table 5).

**Table 4:** Effect of ruminal inocula and diets on the fermentation parameters *in vitro* (*N* = 9).

**Inoculum**	**Diet**	**pH**	**NH_**3**_-N (mg/L)**	**Gas (mL/g)**	**CH_**4**_ (mM)**	**IVDMD (g/kg DM)**	**VFA mM**	**A mol%**	**P mol%**	**iB mol%**	**nB mol%**	**iV mol%**	**nV mol%**	**nC mol%**
Control	Control	6.84	217	229	2.74	574	46.7	66.8	14.9	0.97	13.9	1.81	1.38	0.28
	Zn	6.92	192	222	2.80	518	43.9	66.5	15.2	0.84	14.3	1.60	1.37	0.25
	Herbs	6.91	214	240	2.88	581	46.6	67.0	15.0	0.85	13.8	1.71	1.36	0.31
	Zn+Herbs	6.87	198	227	2.97	502	46.3	66.8	15.3	0.73	14.0	1.54	1.42	0.33
Zn	Control	6.96	305	233	3.06	341	43.8	65.2	15.3	1.55	13.0	2.79	1.75	0.40
	Zn	6.96	301	218	2.62	325	41.1	64.5	15.6	1.55	13.6	2.70	1.74	0.37
	Herbs	6.91	330	250	3.61	347	45.6	65.3	15.5	1.57	12.8	2.73	1.77	0.34
	Zn+Herbs	6.82	273	231	3.31	306	44.0	65.3	15.8	1.27	13.2	2.41	1.69	0.34
Herbs	Control	6.99	248	218	2.66	525	36.2	66.9	16.1	1.10	11.9	2.16	1.59	0.25
	Zn	6.98	214	198	1.73	492	37.8	65.8	15.9	1.08	13.1	2.11	1.65	0.28
	Herbs	6.95	222	216	2.66	554	41.5	66.8	16.4	1.07	11.9	2.05	1.55	0.26
	Zn+Herbs	6.98	216	211	2.47	515	39.5	66.3	16.2	1.05	12.7	1.95	1.58	0.29
Zn+Herbs	Control	7.06	182	184	2.68	585	35.0	65.7	16.7	1.11	12.8	1.95	1.54	0.22
	Zn	7.05	184	173	2.28	575	32.5	64.7	16.3	1.07	14.1	1.94	1.66	0.23
	Herbs	7.04	204	178	2.76	622	35.9	65.8	16.6	1.08	12.9	1.93	1.57	0.21
	Zn+Herbs	7.06	197	180	2.52	571	34.1	65.0	16.7	1.01	13.6	1.83	1.69	0.23
SEM		0.037	24.4	8.65	0.186	39.7	2.60	0.564	0.364	0.104	0.289	0.199	0.065	0.028
**Significance of the effects:**														
Diet		0.512	0.606	0.046	0.001	0.316	0.256	0.159	0.857	0.211	0.001	0.478	0.771	0.730
Inoculum		0.001	0.001	0.001	0.001	0.001	0.001	0.001	0.001	0.001	0.001	0.001	0.001	0.001
Diet × inoculum		0.457	0.985	0.942	0.299	0.996	0.999	0.999	0.997	0.983	0.877	0.999	0.877	0.600

*IVDMD, In vitro dry matter digestibility; VFA, Volatile fatty acids; A, Acetate; P, Propionate; iB, Iso-butyrate; nB, N-butyrate; iV, Iso-valerate; nV, N-valerate; nC, N-caproate*.

**Table 5:** Effect of ruminal inocula and diets on the protozoal population *in vitro* (*N* = 9).

**Inoculum**	**Diet**	***Dasytricha ruminantium* (n/mL)**	***Isotricha* spp. (n/mL)**	***Polyplastron multivesiculatum* (n/mL)**	***Entodinium* spp. (10^**3**^ /mL)**	**Total protozoa (10^**3**^ /mL)**
Control	Control	850	1,010	330	368	378
	Zn	1,320	1,140	350	336	347
	Herbs	1,450	1,230	480	329	338
	Zn+Herbs	2,510	1,280	570	334	343
Zn	Control	2,290	1,880	580	374	380
	Zn	2,330	2,020	710	401	407
	Herbs	2,430	1,830	710	425	430
	Zn+Herbs	2,750	1,900	690	395	401
Herbs	Control	3,270	800	310	321	326
	Zn	3,730	640	250	292	297
	Herbs	3,204	530	210	314	318
	Zn+Herbs	3,890	520	260	292	297
Zn+Herbs	Control	2,440	1,120	180	313	318
	Zn	2,610	1,620	140	350	357
	Herbs	2,060	1,040	130	327	332
	Zn+Herbs	2,720	1,670	250	329	335
SEM		111.5	81.8	29.0	9.1	9.2
**Significance of the effects:**						
Diet		0.073	0.734	0.563	0.982	0.982
Inoculum		<0.001	<0.001	<0.001	0.004	0.004
Diet × inoculum		0.799	0.933	0.870	0.979	0.982

### Effects of Zinc and Herbal Diets on Ruminal Fermentation and Microbiota in the Lambs

The dietary supplements did not significantly affect (*P* > 0.05) the parameters of ruminal fermentation in the lambs (Table 6). The number of ruminal ciliated protozoa, expressed as counts per gram of wet ruminal content or as counts per gram of dry ruminal content in the lambs did not differ significantly (*P* > 0.05) between the groups (Table 7). The lambs contained three ciliate populations. Nineteen animals (68%) had mixed A-B type populations, consisting of *Polyplastron multivesiculatum* (100% prevalence), *Epidinium ecaudatum caudatum* (84% prevalence), and *Ophryoscolex caudatus tricoronatus* (53% prevalence), seven animals (25%) had A type populations, consisting of *P. multivesiculatum* (100% prevalence) and *O. caudatus tricoronatus* (29% prevalence), and two animals (7%) had B type populations, consisting of *E. ecaudatum caudatum* (100% prevalence) but no *Polyplastron* or *Ophryoscolex*. All animals had *Dasytricha ruminantium, Isotricha intestinalis, I. prostoma*, and *Entodinium* spp.

**Table 6:** Effect of zinc and herbs on the parameters of ruminal fermentation in the lambs (*N* = 7).

**Item**	**Control**	**Zn**	**Herbs**	**Zn+Herbs**	**SD**	***P-*value**
pH	6.84	6.95	6.89	7.05	0.245	0.468
Ammonia (mg/L)	110	129	121	102	38.5	0.629
Methane (mM)	0.376	0.382	0.378	0.381	0.071	0.832
Total VFA (mM)	53.9	42.6	47.2	37.3	13.9	0.191
Acetate (mol%)	69.1	69.1	69.8	68.3	2.04	0.663
Propionate (mol%)	13.8	13.9	14.5	14.5	1.55	0.753
*n*-Butyrate (mol%)	13.5	12.1	11.8	12.5	1.59	0.240
*iso*-Butyrate (mol%)	1.03	1.73	1.10	1.65	0.879	0.356
*n*-Valerate (mol%)	0.914	0.910	0.936	1.03	0.221	0.717
*iso*-Valerate (mol%)	1.33	2.00	1.51	1.77	0.822	0.478
*n-*Caproate (mol%)	0.300	0.267	0.263	0.204	0.119	0.546
A:P	5.10	5.04	4.83	4.77	0.567	0.673

**Table 7:** Effects of zinc and herbs on the number of ruminal ciliated protozoa in the lambs (*N* = 7).

**Genus/treatment**	**Control**	**Zn**	**Herbs**	**Zn+Herbs**	**SD**	***P-*value**
*Dasytricha sp*. (c/g wRC)^a^	4,754	5, 28	6,717	8,619	5,004	0.433
*Isotricha spp*. (c/g wRC)	2,280	3,132	2,656	2,043	1,581	0.417
*Polyplastron spp*. (c/g wRC)	2,571	2,723	2,311	663	1,445	0.064
*Epidinium spp*. (c/g wRC)	15,905	160	3,055	13,612	9,558	0.662
*Entodinium spp*. (c/g wRC)	465,316	485,455	412,898	457,362	93,530	0.392
Total protozoa (c/g wRC)	489,413	500,892	427,987	483,593	95,191	0.454
*Dasytricha sp*. (c/g DM)^b^	492	609	701	904	503	0.529
*Isotricha spp*. (c/g DM)	301	401	327	241	228	0.275
*Polyplastron spp*. (c/g DM)	234	333	291	75	216	0.133
*Epidinium spp*. (c/g DM)	2,258	18	274	1,566	1,123	0.044
*Entodinium spp*. (c/g DM)	63,146	59,933	49,355	52,791	17,298	0.490
Total protozoa (c/g DM)	66,430	61,914	50,987	55,729	17,936	0.400

a Expressed as count (c) per gram of wet ruminal content (wRC);

b *Expressed as count per gram of dry matter (DM) of ruminal content*.

**Figure 1:**
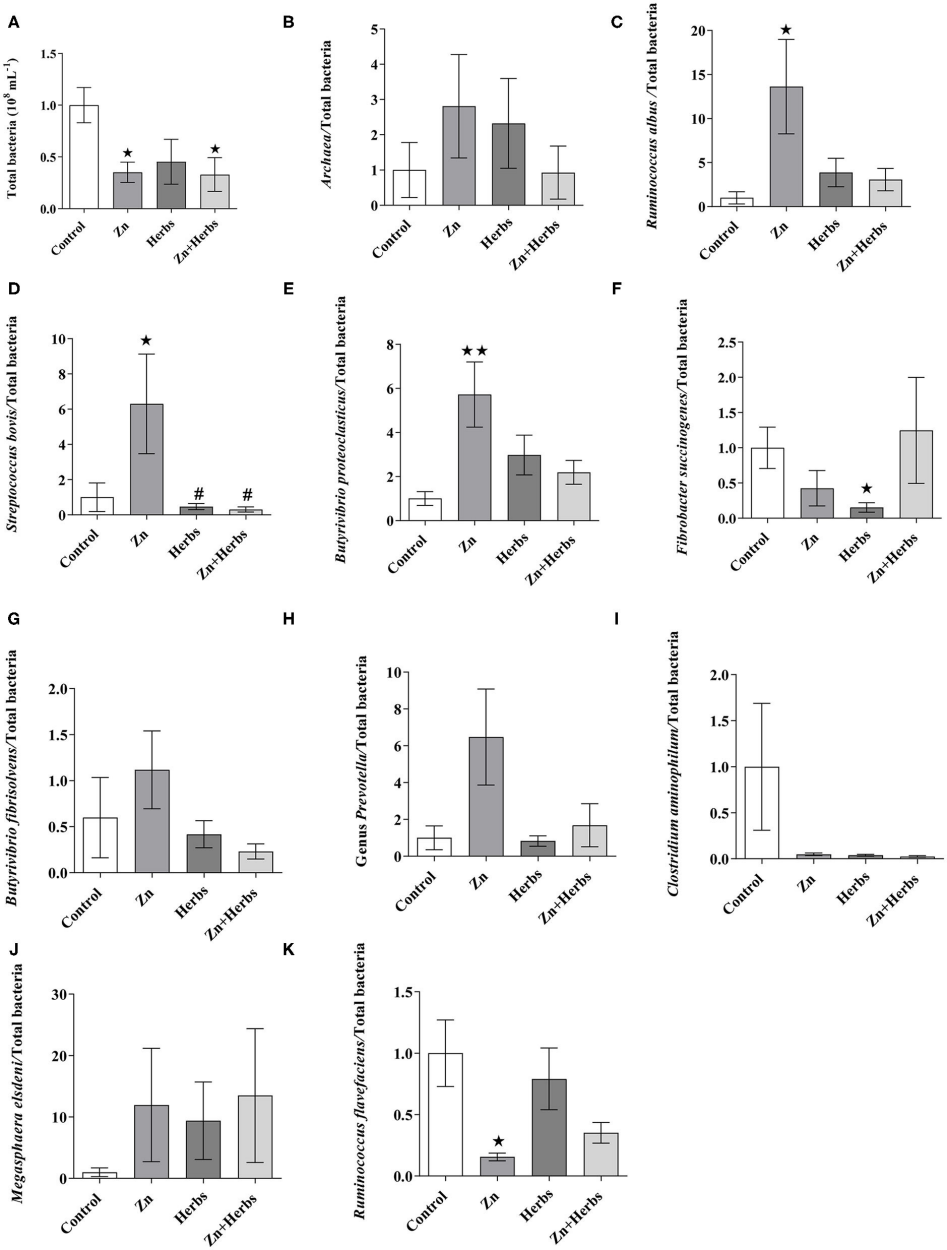
Effects of the Control, Zn, Herbs, and Zn+Herbs diets on the relative abundance of the 16S rRNA gene (expressed relative to the total abundance of bacterial genes in the Control group) of the ruminal bacterial population for **(A)** total bacteria, **(B)** Archaea, **(C)** *Ruminococcus albus*, **(D**) *Streptococcus bovis*, **(E)** *Butyrivibrio proteoclasticus*, **(F)** *Fibrobacter succinogenes*, **(G)** *Butyrivibrio fibrisolvens*, **(H)** *Prevotella*, **(I)** Clostridium *aminophilum*, **(J)** Megasphaera *elsdeni*, and **(K)** *Ruminococcus flavefaciens*. *P < 0.05 and **P < 0.01 relative to the Control group; ^#^*P* < 0.05 relative to the Zn group.

The total bacterial populations (Figure 1A) were significantly lower (*P* < 0.05) for the Zn and Zn+Herbs groups than the Control group, but the relative abundances of *Ruminococcus albus* (Figure 1C), *Streptococcus bovis* (Figure 1D) and *Butyrivibrio proteoclasticus* (Figure 1E) were significantly higher in the group fed the Zn diets. In contrast, the relative abundance of *Ruminococcus flavefaciens* (Figure 1K) was significantly lower (*P* < 0.05) in the Zn than the Control group. The relative abundance of *Fibrobacter succinogenes* (Figure 1F) was significantly lower (*P* < 0.05) in the Herbs than the Control group. The other microbial populations, such as those of *Archaea* (Figure 1B), *Butyrivibrio fibrisolvens* (Figure 1G), *Prevotella* (Figure 1H), Clostridium aminophilum (Figure 1I) and Megasphaera elsdenii (Figure 1J), did not differ significantly (*P* > 0.05) among the groups.

### Hematological Parameters

The count of red blood cells, hemoglobin level and hematocrit were not significantly influenced by time, treatment or the treatment × time interaction (*P* > 0.05) (Table 8). Time significantly affected the mean corpuscular volumes (*P* < 0.001). Treatment and time significantly affected neutrophil levels (*P* < 0.05), and time significantly affected the counts of lymphocytes and eosinophils (*P* < 0.001 and < 0.05, respectively).

**Table 8:** Effects of zinc and herbs on the hematological parameters of the lambs (*N* = 7).

**Item**	**Day**	**Control**	**Zn**	**Herbs**	**Zn+Herbs**	**SD**	**Significance of effects:**		
							**Treatment**	**Time**	**Treatment × time**
Red blood cells (T/L)	0	11.1	10.8	10.8	11.0	0.324			
	35	10.5	10.5	10.7	10.4	0.863	NS	NS	NS
	70	10.1	10.8	10.4	9.47	1.28			
Hemoglobin (g/L)	0	102.8	99.1	96.2	98.0	7.08			
	35	97.8	99.2	101.3	98.6	7.56	NS	NS	NS
	70	82.7	100.0	100.6	98.9	15.8			
Hematocrit (L/L)	0	0.194	0.218	0.214	0.216	0.028			
	35	0.228	0.230	0.229	0.222	0.014	NS	NS	NS
	70	0.226	0.236	0.229	0.208	0.025			
Mean corpuscular volume (fL)	0	20.0	20.3	19.9	19.8	1.15			
	35	21.8	21.8	21.6	21.5	1.28	NS	***	NS
	70	22.6	22.0	22.0	22.1	1.14			
Total leukocytes (g/L)	0	8.26	8.70	7.67	6.60	2.34			
	35	8.33	8.45	8.25	7.70	1.69	NS	NS	NS
	70	8.55	6.54	8.83	8.59	1.33			
Neutrophils (g/L)	0	2.60	3.90	2.56	2.09	1.36			
	35	2.79	3.00	3.03	2.37	0.897	NS	NS	*
	70	3.11	2.01	4.04	3.26	0.953			
Lymphocytes (g/L)	0	3.14	2.50	2.44	2.37	0.903			
	35	3.54	3.51	3.27	3.68	1.41	NS	*	NS
	70	2.96	3.00	2.76	3.03	0.894			
Monocytes (g/L)	0	2.29	1.76	2.41	1.72	0.715			
	35	1.75	1.53	1.61	1.39	0.667	NS	NS	NS
	70	2.18	1.20	1.77	1.94	0.766			
Eosinophils (g/L)	0	0.052	0.044	0.036	0.042	0.019			
	35	0.084	0.150	0.112	0.120	0.079	NS	***	NS
	70	0.056	0.098	0.065	0.092	0.051			
Basophils (g/L)	0	0.257	0.499	0.228	0.370	0.396			
	35	0.170	0.258	0.074	0.132	0.154	NS	NS	NS
	70	0.239	0.221	0.199	0.267	0.207			

* P < 0.05, **P < 0.01,

*** *P < 0.001*.

### Effects of Diet on the Morphological Parameters of the Ruminal Papillae

**Figure 2:**
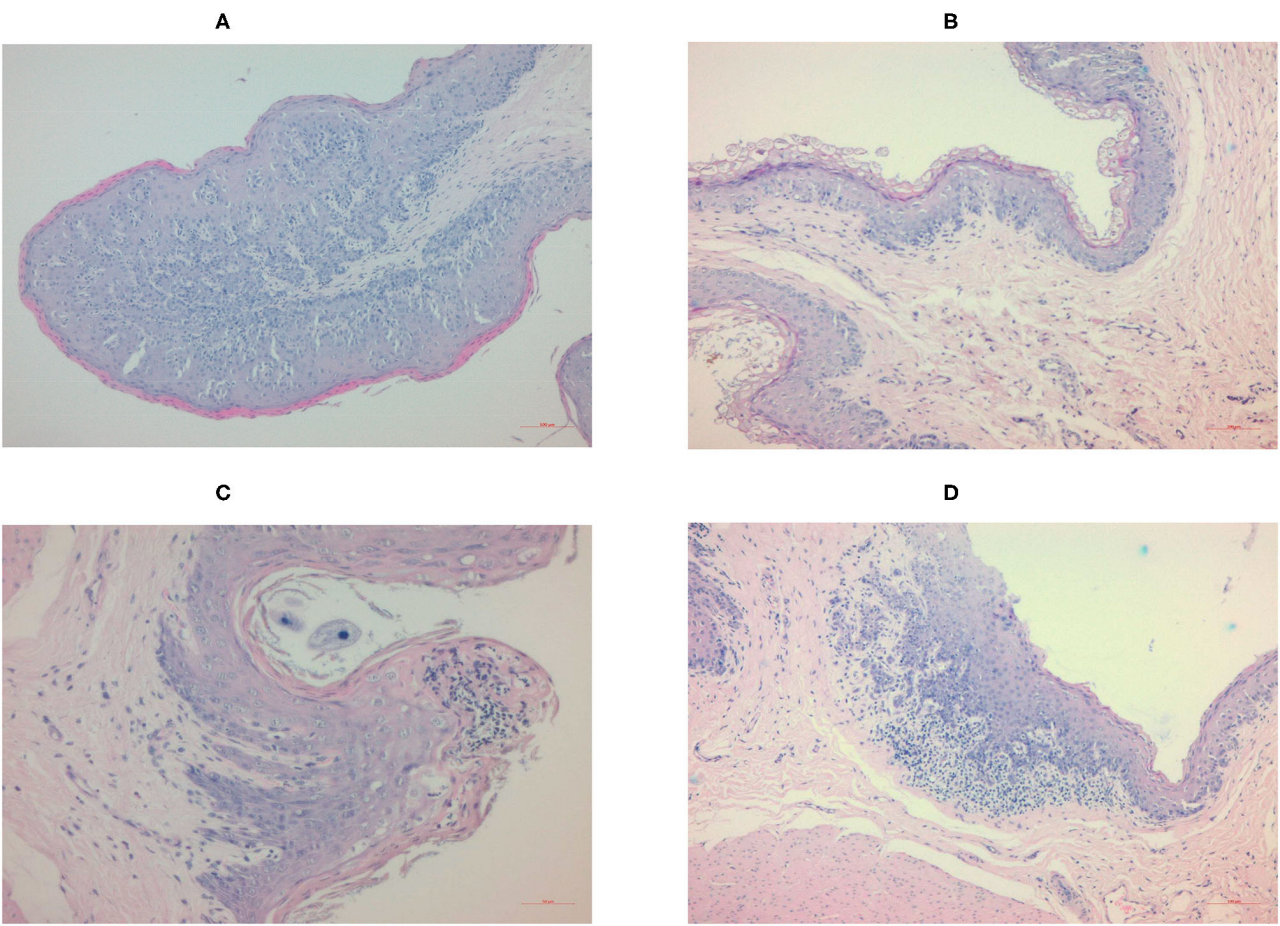
Histology of the ruminal papillae of the lambs in the **(A)** Control, **(B)** Zn, **(C)** Herbs, and **(D)** Zn+Herbs groups. The papillae are stained with hematoxylin-eosin and viewed using a 10 × objective lens.

The histology of the ruminal papillae of the Control and Zn groups were normal (Figures 2A,B). The sizes of the papillae varied in the Herbs group (Figure 2C), and both the type and number of keratinized epithelial cells varied in the Zn+Herbs group (Figure 2D). A focally mixed infiltration of inflammatory cells in individual papillae within the epithelial layer was observed in two animals fed the Herbs diet and within the lamina propria in most animals fed the Zn+Herbs diet.

## Discussion

Our previous results indicated that the addition of zinc to the diet of lambs did not negatively influence microbial activity in the rumen or large intestine (4). A mixture of herbs containing FO, MS, AA, and MC also possessed strong ruminal antioxidant capacity and reduced gastrointestinal concentrations of methane and ammonia *in vitro* (15). To the best of our knowledge, however, the present experiment is the first to determine the interactions between supplementations with organic zinc and herbs by combining phytochemical, physiological, microbiological and histopathological measurements in the rumen of lambs.

The phytochemical substances in FO consisted of flavonoids, alkaloids and phenolic acids. There were mainly quercetin-O-Hex-Hex (2.384 g/kg DM), quercetin O-Pen-Hex (3.5 g/kg DM), fumariline (1.728 g/kg DM), fumaricine (0.102 g/kg DM), and caffeoylmalic acid (1.212 g/kg DM). The flavonoid (flavonol) quercetin possesses various antioxidative and anti-inflammatory effects and metabolic health-promoting properties (28). Both isoquinoline alkaloids fumariline and fumaricine contribute to the important pharmacological activities of FO (29). The phenolic compound caffeoylmalic acid can protect protein from degradation in ruminants that use forage protein (30). The concentrations of the flavonoids, delphinidin 5-glucoside 3-lathyroside (1.644 g/kg DM) and apigenin-O-Hex (1.56 g/kg DM), were highest in MS. The health-promoting effect of apigenin O-Hex, which has therapeutic potential, has been reported (31), but flavonoids generally possess beneficial biochemical properties with predominantly protective roles against many diseases (32). The phytochemical substances in AA consisted mainly of phenolic acids, including chlorogenic acid (3.416 g/kg DM) and 1,5-dicaffeoylquinic acid (2.124 g/kg DM), which possess antibacterial, anthelmintic, anti-inflammatory and antioxidant biological activities *in vitro* and *in vivo* (33, 34). Similarly, MC contained phenolic acids (12.084 g/kg DM), mainly methyl 4-O-beta-d-glucopyranosylcaffeate (3.202 g/kg DM), with well-known antioxidant activity (35), and contained derivatives of caffeoylquinic acid, which have anti-inflammatory biological activities (36).

The mixture of the dry medicinal herbs had high concentrations of flavonoids, especially quercetin (7.6 mg/g DM) (15). Quercetin, after intraruminal application (10 and 50 mg/kg BW) in cows, is a flavonoid extensively degraded by ruminal microbiota without negative effects on ruminal fermentation (37). The administration of some flavonoids with antimicrobial properties, however, can affect the gastrointestinal microbiota. The Zn, Herbs and Zn+Herbs diets under *in vitro* conditions in our study only affected the levels of total gas, methane and *n*-butyrate. The reported effects of flavonoids as potential dietary additives for ruminants (e.g., quercetin, myricetin, kaempferol, and rutin) have been inconsistent, due mainly to their potential antimicrobial effects (38–40).

The *in vitro* ruminal inocula (Table 5), however, significantly affected all parameters of fermentation and species of protozoa. The effects of the inocula could be ascribed to the diverse ruminal ciliate populations and companion bacterial populations in the animals. Table 5 presents the number of protozoa in each group. Not all treatment groups, however, contained *Ophryoscolex* and *Epidinium*. The mixed A-B type ciliate population was also prevalent (19 animals). This finding probably indicates a gradual change from the B type population to the dominant A type population in the lambs, because *Polyplastron* feeds on *Epidinium* until it disappears from the ciliate population (25). The inocula had significant effects, mainly on *Dasytricha* species, probably caused by the long-term dietary supplementation with zinc or herbs, which can influence the composition of the eubacterial community and the enzymatic activities of ruminal microorganisms, especially amylolytic and cellulolytic enzymes (41, 42).

The Zn, Herbs and Zn+Herbs diets did not significantly affect the parameters of fermentation or the protozoal populations in the lambs. This finding probably indicates relatively low contents of antimethanogenic phytochemical substances or the adaptation of the microbiota to both herbal (43, 44) and/or zinc (45) diets. Herbal diets can influence the ruminal microbiome, the kinetics of fermentation and the response and adaptation to antimethanogenic compounds and diets that sometimes lead to the inconsistent efficacies of phytochemical substances (46). Zn is also involved in a wide assortment of physiological processes, so nutrient digestibility may be affected by supplemental Zn, which is incorporated into enzymes throughout the body and is critical for most metabolic processes in ruminants (47). Zinc in the diet of ruminants can substantially influence ruminal fermentation (48, 49). Low doses of zinc (20–70 mg Zn/kg diet) only weakly affect ruminal fermentation (4, 10), but higher doses (250–1,142 mg Zn/kg diet) can affect ruminal protozoal populations and protein degradation (50). The beneficial effects of zinc (i.e., its antioxidant, anti-inflammatory and antiapoptotic properties) strongly depend on both the source and concentration of the zinc, even though zinc retention in lambs can be similar regardless of these factors (47), and too much or too little zinc in diets can have the opposite effect (51, 52).

The amount of starch in all our diets was similar, so the diets probably similarly influenced the efficiency of the growth of the majority of the ruminal ciliates (53). Total bacteria, however, were lower in the Zn and Zn+Herbs groups than the Control group. The relative abundances of the cellulolytic bacterium *R. albus*, the amylolytic bacterium *S. bovis* and the polysaccharide-degrading bacterium *B. proteoclasticus* were higher, and the abundance of the cellulolytic bacterium *R*. flavefaciens was lower in the Zn group than the other groups. Some bacterial species were probably enriched by Zn supplementation at the expense of total bacterial abundance (45). These changes in total bacteria and the relative abundance of some bacteria in the Zn group were not accompanied by changes in VFAs in ruminal fermentation *in vivo*. This finding indicates a direct effect on ruminal microbiota due to an interaction with crude protein rather than to the benefits of Zn supplementation, which is exerted solely on the host organism (8). The lower relative abundance of cellulolytic bacteria (i.e., *F. succinogenes* and *R. flavefaciens*) in the Zn or Zn+Herbs groups, respectively, however, probably also lowered the digestibility of the substrate *in vitro* in these groups (Table 4). The ruminal microbiota may have a specific requirement for zinc supplementation that does not cause a major shift in the ruminal bacterial community and does not have negative consequences for digestion or animal health (45). Some bacterial phylotypes can also contribute to differences in feed efficiency and host productivity and can or need not depend on the diet (54). The relative abundance of the starch-fermenting bacterium *S. bovis* was lower in the Herbs and Zn+Herbs groups than the Zn group, but the relative abundance of the cellulolytic bacterium *F. succinogenes* was lower in the Herbs and Zn+Herbs groups than the Control group, probably due to the antimicrobial activity of some flavonoids, which can increase competition among bacteria (40, 55).

Ruminal VFAs are absorbed through the ruminal epithelium, and the rate of absorption depends on the VFA concentration, the surface area of the ruminal papillae and the availability of transport proteins (56, 57). The ruminal papillae of the lambs in the Control and Zn groups were histologically normal, with diverse sizes of the papillae and the type and number of keratinized epithelial cells. The external layer of vesiculated keratinized cells of the ruminal epithelium is an absorption barrier to the transport of molecules from the rumen to the blood (58). The stratum corneum in the Zn group contained several layers of vacuolated horn cells, with a large amount of keratin in the cytoplasm and cellular organelles. The lambs in the Herbs and Zn+Herbs groups had ruminal papillae with diverse histological structures, mostly the size of the papillae and type and number of keratinized epithelial cells. Butyrate stimulates the development of ruminal papillae (59), but the *in vitro* molar proportion of *n*-butyrate was higher only in the Zn and Zn+Herbs groups. The amount of ruminal VFA absorption can decrease as ruminal parakeratosis increases (60), and a physical barrier could reduce the transport of VFAs to the deepest layers of the epithelium. The health of the ruminal epithelium probably deteriorated because the infiltration of inflammatory cells in individual papillae in the epithelial layer and the lamina propria was focally mixed in the lambs in the Herbs and Zn+Herbs groups, respectively. The hematological parameters of the lambs, however, were not affected by the treatments. The application of the Herbs treatment for 70 d may have been too long.

Nutraceuticals provide health benefits beyond basic nutrition. The vast number of naturally occurring health-enhancing substances are of herbal origin, but many physiologically active components, such as trace elements, also play important roles in the promotion of animal health. Limited information is available on the effects of nutraceuticals such as zinc and/or herbs on blood profiles (16, 61). Pharmacological and clinical studies suggest that *M. sylvestris, A. absinthium* and *M. chamomilla* are promising herbs for the treatment of gastrointestinal disorders (62). Dietary supplementation with *A. absinthium* can also enhance the rate of growth of lambs, thereby increasing weight gains (63). Different nutrients, however, may generally improve the absorptive capability of the ruminal epithelium, protect the epithelium against damage and alter the expression of genes regulating ruminal epithelial morphology (64–66).

## Conclusions

The ability of dietary supplementation with organic zinc (70 mg Zn/kg diet) and herbs (100 g DM/d) to influence ruminal fermentation and the composition of ruminal microbiota *in vitro* was not confirmed *in vivo*. The dietary supplements did not significantly affect the parameters of ruminal fermentation or the protozoal population of the lambs, probably because the lambs adapted to the diets during the 70-d feeding, with lower total bacteria and a shift in the relative abundances of cellulolytic and amylolytic bacteria in the Zn group. Our results, however, also indicated that long-term dietary supplementation with organic zinc combined with a mixture of medicinal herbs could negatively affect the health of the ruminal epithelium. More *in vivo* experiments are therefore necessary.

## Data Availability Statement

The original contributions presented in the study are included in the article/supplementary material, further inquiries can be directed to the corresponding author/s.

## Ethics Statement

The animal study was reviewed and approved by the Ethical Committee of the Institute of Animal Physiology of Centre of Biosciences of SAS approved the experimental protocol (resolution number Ro-3355/16-221).

## Author Contributions

DP, DM, and KK: resources, formal analysis, and investigation. PK, HH, and DM: investigation, methodology, and software. SK: investigation and methodology. AL: histological analyses. SS: bioactive compound analysis. AC and MS-S: conceptualization and data curation. KC: project administration, funding acquisition, and supervision. ZV: validation, writing—review, and editing. All authors read and approved the final manuscript.

## Conflict of Interest

The authors declare that the research was conducted in the absence of any commercial or financial relationships that could be construed as a potential conflict of interest.

## References

[1] Matthews C Crispie F Lewis E Reid M O'Toole PW Cotter PD. The rumen microbiome: a crucial consideration when optimising milk and meat production and nitrogen utilisation efficiency. Gut microbes. (2019) 10:115–32. 10.1080/19490976.2018.1505176 30207838 PMC6546327

[2] Newbold CJ Ramos-Morales E. Review: Ruminal microbiome and microbial metabolome: effects of diet and ruminant host. Animal. (2020) 1:78–86. 10.1017/S1751731119003252 32024572

[3] Wencelová M Váradyová Z Mihaliková K Cobanová K Plachá I Pristaš P. Rumen fermentation pattern, lipid metabolism and the microbial community of sheep fed a high-concentrate diet supplemented with a mix of medicinal plants. Small Rumin Res. (2015) 125:64–72. 10.1016/j.smallrumres.2015.01.028

[4] Váradyová Z Mravčáková D Holodová M Grešáková L Pisarčíková J Barszcz M. Modulation of ruminal and intestinal fermentation by medicinal plants and zinc from different sources. J Anim Physiol Anim Nutr. (2018) 102:1131–45. 10.1111/jpn.12940 29901842

[5] Mravčáková D Váradyová Z Kopčáková A Cobanová K Grešáková L Kišidayová S. Natural chemotherapeutic alternatives for controlling of haemonchosis in sheep. BMC Vet. Res. (2019) 15:302. 10.1186/s12917-019-2050-2 31429752 PMC6700814

[6] Mravčáková D Komáromyová M Babják M Urda Dolinská M Königová A Petrič D. Anthelmintic activity of wormwood (*Artemisia absinthium* L.) and mallow (*Malva sylvestris* L.) against *Haemonchus contortus* in sheep. Animals. (2020) 10:219. 10.3390/ani10020219 32013192 PMC7070545

[7] Ibrahim KS El-Sayed EM. Potential role of nutrients on immunity. Int Food Res J. (2016) 23:464–74.

[8] Hilal EY Elkhairey MAE Osman AOA. The role of zinc, manganese and copper in rumen metabolism and immune function: a review article. Open J Anim Sci. (2016) 6:304–24. 10.4236/ojas.2016.64035

[9] Spears JW. Trace mineral bioavailability in ruminants. J Nutr. (2003) 133:1506S−9. 10.1093/jn/133.5.1506S 12730454

[10] Spears JW Schlegel P Seal MC Lloyd KE. Bioavailability of zinc from zinc sulfate and different organic zinc sources and their effects on ruminal volatile fatty acid proportions. Livest Prod Sci. (2004) 90:211–7. 10.1016/j.livprodsci.2004.05.001

[11] EFSA. Scientific Opinion on the potential reduction of the currently authorised maximum zinc content in complete feed. EFSA J. (2014) 12:3668. 10.2903/j.efsa.2014.3668 PMC13159935 42125380

[12] López-Alonso M. Trace minerals and livestock: not too much not too little. ISRN Vet Sci. (2012) 2012:704825. 10.5402/2012/704825 23762589 PMC3671743

[13] Graham C Simmons NL. Functional organization of the bovine rumen epithelium. Am J Physiol Regul Integr Comp Physiol. (2005) 288:R173–81. 10.1152/ajpregu.00425.2004 15319221

[14] Moens E Veldhoen M. Epithelial barrier biology: good fences make good neighbours. Immunology. (2012) 135:1–8. 10.1111/j.1365-2567.2011.03506.x 22044254 PMC3246647

[15] Petrič D Mravčáková D Kucková K Cobanová K Kišidayová S Cieslak A. Effect of dry medicinal plants (wormwood, chamomile, fumitory and mallow) on *in vitro* ruminal antioxidant capacity and fermentation patterns of sheep. J Anim Physiol Anim Nutr. (2020) 104:1219–32. 10.1111/jpn.13349 32202350

[16] Váradyová Z Mravčáková D Babják M Bryszak M Grešáková L Cobanová K. Effects of herbal nutraceuticals and/or zinc against *Haemonchus contortus* in lambs experimentally infected. BMC Vet Res. (2018) 14:78. 10.1186/s12917-018-1405-4 29523134 PMC5845177

[17] European Commission (EC). Council Regulation (EC) 1099/2009 of 24 September 2009 on the protection of animals at the time of killing. Off J Eur Union. (2009) L303:1–30.

[18] Yánez-Ruiz DR Bannink A Dijkstra J Kebreab E Morgavi DP O'Kiely P. Design, implementation and interpretation of *in vitro* batch culture experiments to assess enteric methane mitigation in ruminants—a review. Anim Feed Sci Technol. (2016) 216:1–18. 10.1016/j.anifeedsci.2016.03.016

[19] McDougall EI. Studies on ruminant saliva. I. The composition and output of sheep's saliva. Biochem J. (1948) 43:99–109. 16748377 PMC1274641

[20] Association of Official Analytical Chemists. Official Methods of Analysis of AOAC International. Washington, DC (2000).

[21] Van Soest PJ Robertson JB Lewis BA. Methods for dietary fiber neutral detergent fiber, and non-starch polysaccharides in relation to animal nutrition. J Dairy Sci. (1991) 74:3583–97. 10.3168/jds.S0022-0302(91)78551-2 1660498

[22] Wencelová M Váradyová Z Pristaš P Cobanová K Plachá I Kišidayová S. Effects of diet supplementation with herbal blend and sunflower seeds on fermentation parameters, microbial population, and fatty acid profile in rumen of sheep. Czech J Anim Sci. (2016) 61:551–9. 10.17221/17/2016-CJAS

[23] Moss AR Jouany JP Newbold J. Methane production by ruminants: its contribution to global warming. Ann Zootech. (2000) 49:231–53. 10.1051/animres:2000119

[24] Broderick GA Kang JH. Automated simultaneous determination of ammonia and total amino acids in ruminal fluid and *in vitro* media. J Dairy Sci. (1980) 63:64–75. 10.3168/jds.S0022-0302(80)82888-8 7372898

[25] Williams AG Coleman GS. The Rumen Protozoa. New York, NY: Springer Verlag (1992).

[26] Henderson G Cox F Kittelmann S Miri VH Zethof M Noel SJ. Effect of DNA Extraction methods and sampling techniques on the apparent structure of cow and sheep rumen microbial communities. PLoS ONE. (2013) 8:e74787. 10.1371/journal.pone.0074787 24040342 PMC3770609

[27] Stevenson DM Weimer PJ. Dominance of *Prevotella* and low abundance of classical ruminal bacterial species in the bovine rumen revealed by relative quantification real-time PCR. Appl Microbiol Biotechnol. (2007) 75:165–74. 10.1007/s00253-006-0802-y 17235560

[28] Erlund I. Review of the flavonoids quercetin, hesperetin, and naringenin. Dietary sources, bioactivities, bioavailability, and epidemiology. Nutr Res. (2004) 24:851–74. 10.1016/j.nutres.2004.07.005

[29] Al-Snafi AE. Constituents and pharmacology of *Fumaria officinalis*- A review. IOSR J Pharm. (2020) 10:17–25.

[30] Sullivan ML Zeller WE. Efficacy of various naturally occurring caffeic acid derivatives in preventing post-harvest protein losses in forages. J Sci Food Agric. (2013) 93:219–26. 10.1002/jsfa.5781 22777944

[31] Salehi B Venditti A Sharifi-Rad M Kregiel D Sharifi-Rad J Durazzo A. The therapeutic potential of apigenin. Int J Mol Sci. (2019) 20:1305. 10.3390/ijms20061305 30875872 PMC6472148

[32] Kumar S Pandey AK. Chemistry and biological activities of flavonoids: an overview. Sci World J. (2013) 2013:162750. 10.1155/2013/162750 24470791 PMC3891543

[33] Sato Y Itagaki S Kurokawa T Ogura J Kobayashi M Hirano T. *In vitro* and *in vivo* antioxidant properties of chlorogenic acid and caffeic acid. Int J Pharm. (2011) 403:136–8. 10.1016/j.ijpharm.2010.09.035 20933071

[34] Tajik N Tajik M Mack I Enck P. The potential effects of chlorogenic acid, the main phenolic components in coffee, on health: a comprehensive review of the literature. Eur J Nutr. (2017) 56:2215–44. 10.1007/s00394-017-1379-1 28391515

[35] Braham H Mighri Z Jannet HB Matthew S Abreu PM. Antioxidant phenolic glycosides from *Moricandia arvensis*. J Nat Prod. (2005) 68:517–22. 10.1021/np049581m 15844940

[36] Santos MD Chen G Almeida MC Soares DM de Souza GE Lopes NP. Effects of caffeoylquinic acid derivatives and C-flavonoid from *Lychnophora ericoides* on *in vitro* inflammatory mediator production. Nat Prod Commun. (2010) 5:733–40. 20521538 PMC2913437

[37] Berger LM Blank R Zorn F Wein S Metges CC Wolffram S. Ruminal degradation of quercetin and its influence on fermentation in ruminants. J Dairy Sci. (2015) 98:5688–98. 10.3168/jds.2015-9633 26094220

[38] Broudiscou L-P Lassalas B. Effects of *Lavandula officinalis* and *Equisetum arvense* dry extracts and isoquercitrin on the fermentation of diets varying in forage contents by rumen microorganisms in batch culture. Reprod Nutr Dev. (2000) 40:431–40. 10.1051/rnd:2000110 11140815

[39] Bodas R López S Fernández M García-González R Rodríguez AB Wallace RJ. *In vitro* screening of the potential of numerous plant species as antimethanogenic feed additives for ruminants. Anim Feed Sci Technol. (2008) 145:245–58. 10.1016/j.anifeedsci.2007.04.015

[40] Oskoueian E Norhani A Oskoueian A. Effects of flavonoids on rumen fermentation activity, methane production, and microbial population. Biomed Res Int. (2013) 2013:8. 10.1155/2013/349129 24175289 PMC3794516

[41] Cieslak A Zmora P Pers-Kamczyc E Szumacher-Strabel M. Effects of tannins source (*Vaccinium vitis idaea* L.) on rumen microbial fermentation *in vivo*. Anim Feed Sci Technol. (2012) 176:102–6. 10.1016/j.anifeedsci.2012.07.012

[42] Kišidayová S Pristaš P Zimovčáková M Blanár Wencelová M Homolová L Mihaliková K. The effects of high dose of two manganese supplements (organic and inorganic) on the rumen microbial ecosystem. PLoS ONE. (2018) 13:e0191158. 10.1371/journal.pone.0191158 29324899 PMC5764370

[43] Patra AK Saxena J. A new perspective on the use of plant secondary metabolites to inhibit methanogenesis in the rumen. Phytochemistry. (2010) 71:1198–222. 10.1016/j.phytochem.2010.05.010 20570294

[44] Patra AK Yu Z. Effects of adaptation of *in vitro* rumen culture to garlic oil, nitrate, and saponin and their combinations on methanogenesis, fermentation, and abundances and diversity of microbial populations. Front Microbiol. (2015) 6:1434. 10.3389/fmicb.2015.01434 26733975 PMC4686681

[45] Ishaq SL Page CM Yeoman CJ Murphy TW Van Emon ML Stewart WC. Zinc AA supplementation alters yearling ram rumen bacterial communities but zinc sulfate supplementation does not. J Anim Sci. (2019) 97:687–97. 10.1093/jas/sky456 30508094 PMC6358250

[46] Patra A Park T Kim M Yu Z. Rumen methanogens and mitigation of methane emission by anti-methanogenic compounds and substances. J Anim Sci Biotechnol. (2017) 8:13. 10.1186/s40104-017-0145-9 28149512 PMC5270371

[47] VanValin KR Genther-Schroeder ON Carmichael RN Blank CP Deters EL Hartman SJ. Influence of dietary zinc concentration and supplemental zinc source on nutrient digestibility, zinc absorption, and retention in sheep. J Anim Sci. (2018) 96:5336–44. 10.1093/jas/sky384 30299509 PMC6276585

[48] Wang RL Liang JG Lu L Zhang LY Li SF Luo XG. Effect of zinc source on performance, zinc status, immune response, and rumen fermentation of lactating cows. Biol Trace Elem Res. (2013) 152:16–24. 10.1007/s12011-012-9585-4 23279942

[49] Chen M Xi Y Zhang L Zeng H Li Y Han Z. Effects of zinc-bearing palygorskite on rumen fermentation *in vitro*. Asian-Aust J Anim Sci. (2019) 32:63–71. 10.5713/ajas.17.0920 29747497 PMC6325408

[50] Froetschel MA Martin AC Amos HE Evans JJ. Effects of zinc sulfate concentration and feeding frequency on ruminal protozoal numbers, fermentation patterns and amino acid passage in steers. J Anim Sci. (1990) 68:2874–84. 10.2527/1990.6892874x 2211417

[51] Maret W Sandstead HH. Zinc requirements and the risks and benefits of zinc supplementation. J Trace Elem Med Biol. (2006) 20:3–18. 10.1016/j.jtemb.2006.01.006 16632171

[52] Maret W. Zinc biochemistry: from a single zinc enzyme to a key element of life. Adv Nutr. (2013) 4:82–91. 10.3945/an.112.003038 23319127 PMC3648744

[53] Newbold CJ de la Fuente G Belanche A Ramos-Morales E McEwan NR. The role of ciliate protozoa in the rumen. Front Microbiol. (2015) 6:1313. 10.3389/fmicb.2015.01313 PMC4659874 26635774

[54] Hernandez-Sanabria E Goonewardene LA Wang Z Durunna ON Moore SS Guan LL. Impact of feed efficiency and diet on adaptive variations in the bacterial community in the rumen fluid of cattle. Appl Environ Microbiol. (2012) 78:1203–14. 10.1128/AEM.05114-11 22156428 PMC3273029

[55] De Nardi R Marchesini G Li S Khafipour E Plaizier KJ Gianesella M. Metagenomic analysis of rumen microbial population in dairy heifers fed a high grain diet supplemented with dicarboxylic acids or polyphenols. BMC Vet Res. (2016) 12:29. 10.1186/s12917-016-0653-4 26896166 PMC4759956

[56] Bannink A France J Lopez S Gerrits WJ Kebreab E Tamminga S. Modeling the implications of feeding strategy on rumen fermentation and functioning of the rumen wall. Anim Feed Sci Technol. (2008) 143:3–26. 10.1016/j.anifeedsci.2007.05.002

[57] Melo LQ Costa SF Lopes F Guerreiro MC Armentano LE Pereira MN. Rumen morphometrics and the effect of digesta pH and volume on volatile fatty acid absorption. J Anim Sci. (2013) 91:1775–83. 10.2527/jas.2011-4999 23345561

[58] Dobson MJ Brown WCB Dobson A Phillipson AT. A histological study of the organization of the rumen epithelium of sheep. Quart J Exptl Physiol. (1956) 41:247–53. 10.1113/expphysiol.1956.sp001186 13485339

[59] Mentschel J Leiser R Mülling C Pfarrer C Claus R. Butyric acid stimulates rumen mucosa development in the calf mainly by a reduction of apoptosis. Arch Tierernahr. (2001) 55:85–102. 10.1080/17450390109386185 12068484

[60] Hinders RG Owen FG. Relation of ruminal parakeratosis development to volatile fatty acid absorption. J Dairy Sci. (1965) 48:1069–74. 10.3168/jds.S0022-0302(65)88393-X 5891234

[61] Mandal GP Dass RS Varshney VP Mondal AB. Effect of zinc supplementation from inorganic and organic sources on growth and blood biochemical profile in crossbred calves. J Anim Feed Sci. (2008) 17:147–56. 10.22358/jafs/66478/2008

[62] Mayer M Vogl CR Amorena M Hamburger M Walkenhorst M. Treatment of organic livestock with medicinal plants: a systematic review of European ethnoveterinary research. Forsch Komplementmed. (2014) 21:375–86. 10.1159/000370216 25592949

[63] Beigh YA Mir DM Ganai AM Ahmad HA Muzamil S. Body biometrics correlation studies on sheep fed wormwood (*Artemisia absinthium* L.) herb supplemented complete diets. Indian J Vet Res. (2019) 28:8–13. 10.5958/0974-0171.2019.00002.5

[64] Wang B Wang D Wu X Cai J Liu M Huang X. Effects of dietary physical or nutritional factors on morphology of rumen papillae and transcriptome changes in lactating dairy cows based on three different forage-based diets. BMC Genomics. (2017) 18:353. 10.1186/s12864-017-3726-2 28477620 PMC5420399

[65] Garcia Diaz T Ferriani Branco A Jacovaci FA Cabreira Jobim C Bolson DC Pratti Daniel JL. Inclusion of live yeast and mannan-oligosaccharides in high grain-based diets for sheep: Ruminal parameters, inflammatory response and rumen morphology. PLoS ONE. (2018) 13:e0193313. 10.1371/journal.pone.0193313 29466450 PMC5821403

[66] Jing XP Peng QH Hu R Zou HW Wang HZ Yu XQ. Dietary supplements during the cold season increase rumen microbial abundance and improve rumen epithelium development in Tibetan sheep. J Anim Sci. (2018) 96:293–305. 10.1093/jas/skx032 29385456 PMC6140854

