# Corrigendum: Impact of Zinc and/or Herbal Mixture on Ruminal Fermentation, Microbiota, and Histopathology in Lambs

**DOI:** 10.3389/fvets.2021.660794

**Published:** 2021-02-17

**Authors:** Daniel Petrič, Dominika Mravčáková, Katarína Kucková, Svetlana Kišidayová, Adam Cieslak, Malgorzata Szumacher-Strabel, Haihao Huang, Pawel Kolodziejski, Anna Lukomska, Sylwester Slusarczyk, Klaudia Čobanová, Zora Váradyová

**Affiliations:** ^1^Institute of Animal Physiology, Centre of Biosciences of the Slovak Academy of Sciences, Košice, Slovakia; ^2^Department of Animal Nutrition, Poznan University of Life Sciences, Poznan, Poland; ^3^Department of Animal Physiology, Biochemistry and Biostructure, Poznan University of Life Sciences, Poznan, Poland; ^4^Department of Preclinical Sciences and Infectious Diseases, Poznan University of Life Sciences, Poznan, Poland; ^5^Department of Pharmaceutical Biology and Botany, Wroclaw Medical University, Wroclaw, Poland

**Keywords:** bacteria, ciliated protozoa, hematological profiles, histology, phytochemicals, sheep

In the original article, there was a mistake in Table 2 and Table 3 as published. **In the columns under m/z [M-H]- in all expressions separating numbers from decimals, there should be dots (.) not a slash (/)**. The corrected Tables 2 and 3 appear below.

**Table 2:** Concentrations of the main bioactive compounds in medicinal herbs (g/kg DM).

**RT (min)**	**UV (nm)**	***m/z*[M-H]^**−**^**	**MS^**2**^**	**MS^**2**^ fragments**	**Formula**	**Compounds**	**Flavonoids**	**Phenolic acids**
***Fumaria officinalis***								
7.00	250/326	295.046	179.0338		C_13_H_12_O_8_	Caffeoylmalic acid		1.212
7.80	227/315	163.0395	119.0499		C_9_H_8_O_3_	O-Coumaric acid		0.742
9.00	255/352	625.1398	301.0337		C_27_H_30_O_17_	Quercetin-O-Hex-Hex	2.384	
9.40	255/352	595.1287	301.0339		C_26_H_28_O_16_	Quercetin O-Pen-Hex	3.500	
9.90	252/351	609.1472	300.0279	285	C_27_H_30_O_16_	Isoquercitrin O-Dhex		0.934
10.20	255/354	463.0882	301.0337		C_21_H_20_O_12_	Quercetin O-Hex	1.706	
10.90	221/329	593.1520	285.0397		C_27_H_30_O_15_	Kaempferol-3-O-rutinoside	0.464	
11.50	255/365	639.1561	315.0504		C_28_H_32_O_17_	Isorhamnetin-O-Hex-Hex	0.558	
						Total contents:	12.211	3.961
***Malva sylvestris***								
7.00	523	757.1846	347.0761	329,261,509	C_32_H_39_O_21_	Delphinidin 5-glucoside 3-lathyroside	1.644	
7.90	308	163.0381	119.0502		C_9_H_8_O_3_	Coumarinic acid		0.468
10.00		609.1458	301.0330		C_27_H_31_O_16_	Quercetin-3-O-rutinoside	0.395	
10.20	268/343	447.0928	285.0386		C_21_H_20_O_11_	Kaempferol-O-Hex	0.494	
11.40	268/336	431.0978	269.0435		C_21_H_20_O_10_	Apigenin-O-Hex	1.560	
						Total contents:	6.479	0.654
***Artemisia absinthium***								
4.10	215/325	353.0877	191.0567	179,161,135	C_16_H_18_O_9_	Chlorogenic acid		3.416
11.00		515.1193	353.0867	191,179,135	C_25_H_24_O_12_	1.5-Dicaffeoylquinic acid		2.124
11.20		653.1719	345.0595	330,302	C_29_H_34_O_17_	Spinacetin 3-rutinoside	0.241	
11.70		515.1192	353.0869	173,179,191,155	C_25_H_24_O_12_	4.5-Dicaffeoylquinic acid		0.610
						Total contents:	0.349	6.482
***Matricaria chamomilla***								
4.30	215/300	353.0877	191.0567		C_16_H_18_O_9_	3-O-Caffeoylquinic acid		1.777
9.00	235/290/319	355.1029	193.049	149	C_16_H_20_O_9_	Methyl 4-O-beta-d-glucopyranosyl caffeate		3.202
9.70	255/354	463.0879	301.0337	151	C_21_H_20_O_12_	Quercetin O-Hex	0.199	
10.30	257	447.0920	285.0386		C_21_H_20_O_11_	Kaempferol O-Hex	1.363	
10.70	217/291/325	515.1189	353.0877	179,191	C_25_H_24_O_12_	3.5-Dicaffeoylquinic acid		0.824
11.00	217/291/325	515.1197	353.0869	191,179	C_25_H_24_O_12_	1.5-Dicaffeoylquinic acid		3.016
11.40	266/300	431.0976	269.0434		C_21_H_20_O_10_	Apigenin O-Hex	0.150	
11.70	215/290/325	515.119	353.0868	173,179,191	C_25_H_24_O_12_	4.5-Dicaffeoylquinic acid		0.851
14.40	218/268/339	473.1085	269.0427	406	C_23_H_22_O_11_	Apigenin -O-(Hex-Ac)	0.210	
						Total contents:	2.442	12.084

**Table 3:** Concentrations of the main alkaloids in *Fumaria officinalis* (g/kg DM).

**RT (min)**	**UV (nm)**	***m/z*[M-H]^**−**^**	**MS^**2**^**	**MS^**2**^ fragments**	**Formula**	**Compounds**	**Alkaloids**
7.70	272	354.1366	305.0811	279,233,323,336	C_20_H_19_NO_5_	Parfumine	0.884
8.40	280	328.1572	265.0865	237,297,313,178	C_19_H_21_NO_4_	Cularimine	0.102
8.50	288	370.1678	291.1029	263,352,337	C_21_H_23_NO_5_	Fumaricine	0.102
8.90	285	326.1410	311.1172	277,294,251,178	C_19_H_19_NO_4_	Cheilanthifoline	0.231
9.20	286	354.1360	275.0713	336,247	C_20_H_19_NO_5_	Chelidonine	0.154
9.40	289	398.1621	338.1397	277,323,249	C_22_H_23_NO_6_	Not determined	0.530
10.20	289	354.1362	275.0692	247,293,206	C_20_H_19_NO_5_	Protopine	0.873
10.60	288	354.1727	206.1139	275,311,338,292	C_21_H_23_NO_4_	Protopine type	0.367
10.80	271	352.1193	279.0647	309,321,263,251	C_20_H_17_NO_5_	Fumariline	1.728
11.20	288	324.1230	249.0764	307,277,219,176	C_19_H_17_NO_4_	Stylopine	0.785
						Total contents:	6.015

The authors apologize for this error and state that this does not change the scientific conclusions of the article in any way. The original article has been updated.

